# Divergent evolution of metachronous follicular lymphoma and extranodal marginal zone lymphoma of mucosa‐associated lymphoid tissue from a common precursor

**DOI:** 10.1002/path.6143

**Published:** 2023-06-22

**Authors:** Maria‐Myrsini Tzioni, Andrew Wotherspoon, Zi Chen, Francesco Cucco, Jasmine Makker, Ming‐Qing Du

**Affiliations:** ^1^ Division of Cellular and Molecular Pathology, Department of Pathology University of Cambridge Cambridge UK; ^2^ Histopathology Department The Royal Marsden Hospital London UK; ^3^ Department of Histopathology, Addenbrooke's Hospital Cambridge University Hospitals NHS Foundation Trust Cambridge UK

**Keywords:** *BCL2* translocation, follicular lymphoma, EMZL, clonal evolution, mutational profiling

## Abstract

The translocation t(14;18)(q32:q21)/*IGH::BCL2* occurs at the pre‐B stage of B‐cell development in the bone marrow and is insufficient for malignant transformation, although it leads to the formation of *in situ* follicular B‐cell neoplasia (ISFN). Despite that, the translocation is the genetic hallmark of follicular lymphoma (FL), it occurs infrequently in metachronous/synchronous lymphomas, including extranodal marginal zone lymphoma of mucosa‐associated lymphoid tissue (EMZL), mantle cell lymphoma, and Hodgkin's lymphoma. In each of these scenarios, the two lymphomas often appear to be clonally related by analyses of *IGH::BCL2* and/or rearranged *IG* genes. However, it remains largely unknown whether one lymphoma originates from the other or they develop independently. We studied five cases of metachronous EMZL and FL. In four cases, the two lymphomas were clonally related, as shown by identical *IGH::BCL2* and/or rearranged *IG* genes or shared mutations. There were common and unique mutations between the paired EMZL and FL, indicating that they developed independently from a common premalignant cell population, harbouring *IGH::BCL2* in three cases. Furthermore, case 1 presented with three metachronous FLs, and all of them originated from a common precursor cell population via divergent evolution. Our findings highlight the multi‐malignant potential of *IGH::BCL2*‐positive B‐cells. © 2023 The Authors. *The Journal of Pathology* published by John Wiley & Sons Ltd on behalf of The Pathological Society of Great Britain and Ireland.

## Introduction

The translocation t(14;18)(q32:q21)/*IGH::BCL2* occurs as a consequence of erroneous *VDJ* recombination at the pre‐B stage of B‐cell development in the bone marrow. The translocation causes overexpression of BCL2 but alone is insufficient for malignant transformation. The translocation can be detected by PCR in peripheral blood lymphocytes of healthy individuals, in as high as 60% of those >40 years age [1]. The *IGH::BCL2*‐positive cells act like reactive B‐cells and are capable of undergoing affinity maturation through the germinal centre (GC) reaction, transiting B‐cell follicles and spreading in peripheral lymphoid tissues. Histologically, these translocation‐positive cells form *in situ* follicular B‐cell neoplasia (ISFN) [2, 3, 4, 5]. They clonally expand while undergoing the GC reaction and are thus at risk of acquiring genetic changes, partially due to off‐target somatic hypermutation activities.

Only a very small proportion of individuals with *IGH::BCL2* will eventually develop a lymphoma, with the majority being follicular lymphoma (FL). However, the *IGH::BCL2*‐positive cells may have the potential to develop into more than one lymphoma as the majority of transformed FLs do not progress directly from their preceding FL but originate via divergent evolution from their commonly related *IGH::BCL2*‐positive premalignant cell population [6, 7, 8]. Additionally, cases with FL may present with other synchronous or metachronous lymphoid malignancies, including chronic lymphocytic leukaemia, Hodgkin's lymphoma, extranodal marginal zone lymphoma of the mucosa‐associated lymphoid tissue (EMZL), and histiocytic cell sarcoma [9]. In most of these cases, the clonal relationship between the paired FL and other synchronous/metachronous lymphoid tumour is confirmed, but whether one lymphoma originates from the other or they develop independently remains largely unknown. We investigated five cases of metachronous FL and EMZL by mutation profiling and showed in four cases that the two lymphomas were clonally related but developed independently from a common premalignant B‐cell population.

## Materials and methods

### Patients and clinical data

The use of archival tissues for research was approved by the ethics committees of the institutions involved.

**Figure 1:**
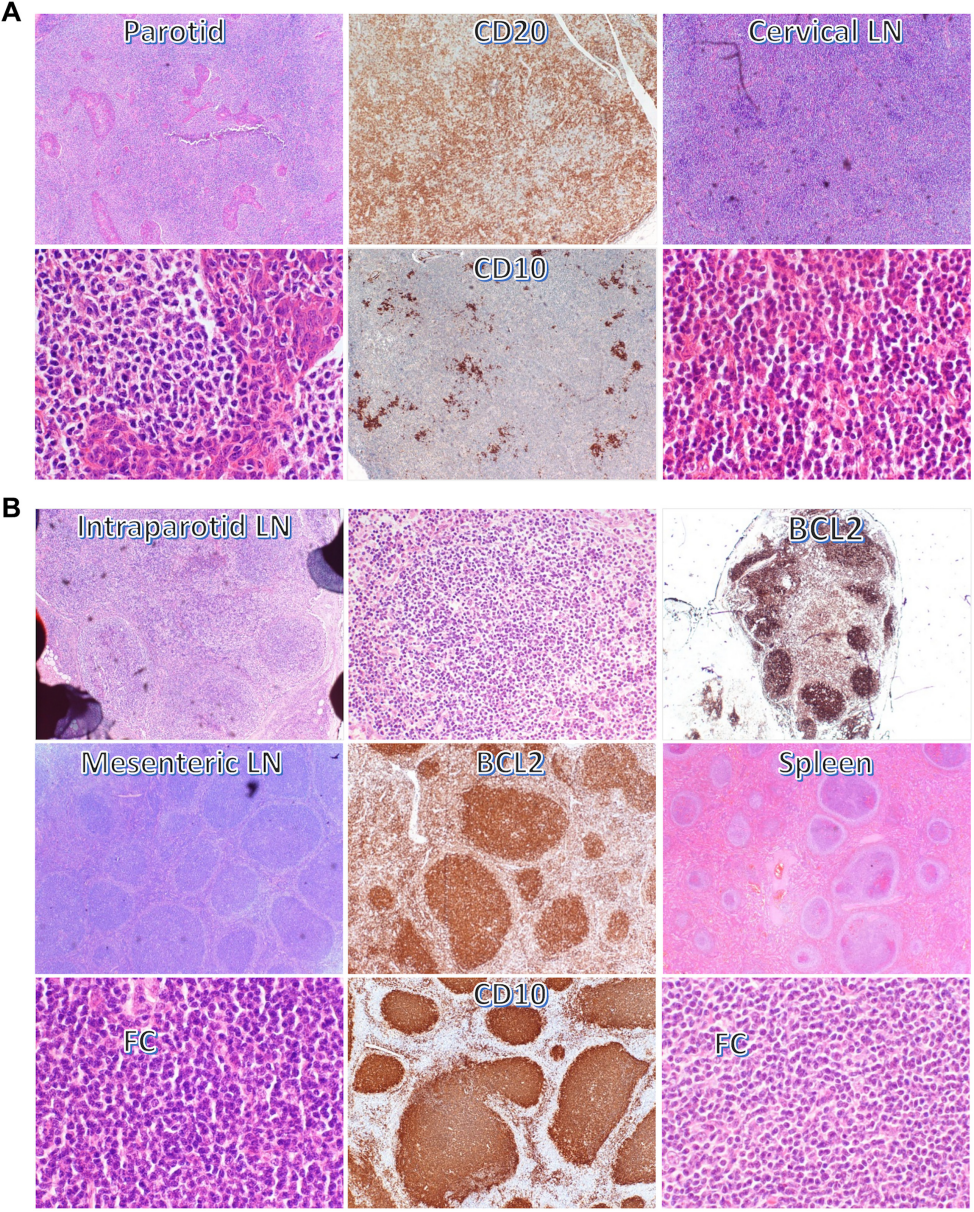
Histological and immunophenotypic features of EMZL and FL in case 1. (A) Parotid biopsies and cervical lymph node (LN) show typical features of EMZL. (B) Intraparotid LN shows strong BCL2 expression in GC B‐cells, loss of zonal polarity, and attenuated mantle zone, while mesenteric LN and spleen display classic features of FL. FC: follicle centre.

Case 1 was the subject of a previous study [10]. The patient was a 44‐year‐old female with a 12‐year history of dry mouth, arthritis of both hands, and swelling of the right parotid gland (Table 1). An initial parotid biopsy and a further parotid biopsy 12 months later showed EMZL (Figure 1A). Eighteen months after the first parotid biopsy the patient presented with enlarged cervical lymph nodes (LNs), with excision biopsy showing involvement by EMZL. Twenty‐four months after the initial parotid biopsy, the patient developed generalised lymphadenopathy and hepatosplenomegaly, and splenectomy was performed together with biopsies of mesenteric and inguinal LNs. All specimens showed classic FL (Figure 1B). There were no systemic treatments for the patient between different biopsies. Previous molecular analyses demonstrated that these different lymphomas harboured an identical *IGH* gene rearrangement and *IGH::BCL2* genomic fusion [10]. Previous histological assessment also identified a small intraparotid LN in the second parotid biopsy, which showed FL (Figure 1B).

**Table 1:** Summary of clinical and laboratory results of cases with metachronous EMZL and FL.

Site biopsy	Diagnosis	Histopathology	Immunophenotype	Molecular data	
**Case 1**: A 44‐year‐old female with 12‐year history of Sjögren's syndrome					
Parotid (E‐bx)	EMZL	Both parotid gland biopsies show diffuse infiltration of centrocyte‐like cells with prominent lymphoepithelial lesions. The neck LN biopsy displays total effacement of its architecture by diffuse centrocyte‐like cells, with remnants of B‐cell follicles	Identical and being: CD20+, CD5−, CD10‐, IgD‐IgM+, Igκ+, Igλ−, BCL2+	*IGH::BCL2* positive by PCR	These different lymphomas harbour an identical *IGH* gene rearrangement and show both common and distinct mutations.
Parotid (E‐bx) (12 months later)					
Neck LN (E‐bx) (18 months later)					
Intraparotid LN, Mesenteric LN, spleen (24 months later) (all E‐bx)	FL1‐2	All these specimens show effacement of normal lymphoid architecture by closely packed follicles with poorly formed mantle	Identical and being: CD20+, CD5−, CD10+, IgD−, IgM+, Igκ+, Igλ−BCL2+	*IGH::BCL2* with same fusion sequence as above	
**Case 2:** A 57‐year‐old female with acute small intestinal obstruction					
Small intestine (ileum) (resection)	EMZL	Diffuse infiltration of small lymphoid cells predominantly in submucosa between B‐cell follicles, with focal invasion to mucosa, and plasmacytoid differentiation	CD20+, CD5−, CD10−, BCL6−, CD23−, CD138−, IgD−, IgM−, Igκ−, Igλ+, BCL2+, Cyclin D1−	*BCL2* trans+ve by FISH	These different lymphomas show an identical clonal pattern with *IGK* (tube B) and both common and distinct mutations.
Peritoneal LN (41 months later) (C‐bx)	FL1‐2	Predominantly small lymphoid cells with vaguely nodular growth pattern over FDC meshworks	CD20+, CD79a+, CD10+, BCL6+, CD5−, CD23−, IgD−, Igκ−, Igλ+, BCL2+, CyclinD1−	*BCL2* trans+ve by FISH	
**Case 3**: A 58‐year‐old male with dysphagia					
Stomach (mucosal bx)	EMZL	Dense infiltrates of small to intermediate‐sized lymphoid cells around glands extending to lamina propria forming lymphoepithelial lesions	CD19+, CD20+, CD5−, CD10−, BCL6−, CD43−, IgD+, IgM+, Igκ−, Igλ+, Cyclin D1−	*MALT1* trans−ve & *BCL6* trans−ve by FISH	Both lymphomas show common and distinct mutations.
Left neck LN (26 months later) (C‐bx)	FL3A	Lymph node architecture effaced by vaguely nodular proliferation of predominantly large lymphoid cells over FDC meshworks	CD19+, CD20+, CD10+, BCL6+, Igκ−, Igλ+, BCL2+	*BCL2* trans−ve and *BCL6* trans−ve by FISH	
**Case 4:** A 55‐year‐old male with bilateral FL of conjunctivae					
Gastric biopsy (mucosal bx)	EMZL	Dense lymphoid infiltrate in mucosa with lymphoepithelial lesions	CD20+, CD79a+, CD10−, BCL6−, CD5−, CD23−, IgD−, IgM−, Igκ+, Igλ−, Cyclin D1−	*BCL2* trans+ve by FISH	All lesions share numerous common mutations, with FL further harbouring subclonal genetic changes.
Left axillary LN (8 days later) (E‐bx)	ISFN	Enlarged node with no specific diagnostic features	Strong BCL2+ in germinal centre B‐cells of several follicles	*BCL2* trans+ve by FISH	
Right buttock soft tissue (17 days later) (C‐bx)	FL1‐2	Dense lymphoid infiltrate, partially nodular, predominantly medium‐sized lymphoid cells with ovoid to centrocytoid nuclei	CD20+, CD79a+, CD10+, BCL6+, IgD−, IgM+, Igκ+, Igλ−, BCL2+	*BCL2* trans+ve by FISH	
**Case 5:** An 82‐year‐old male					
Right thigh subcutaneous mass (C‐bx)	EMZL	Sheets of small to medium‐sized lymphoid cells with ovoid, angulated nuclei, and moderate amount of cytoplasm	CD20+, CD10‐, BCL6−, CD5−, CD23−, Igκ+, Igλ−, BCL2+, Cyclin D1−	n/a	The two lymphomas show different *IGH* rearrangements and distinct mutations without any shared changes.
Right inguinal LN (48 months later) (C‐bx)	FL3A	Vague nodules of medium‐sized to large lymphoid cells, predominantly centrocytes	CD20+, CD79a+, PAX5+, CD10+, BCL6 variably+, CD5−, CD23−, BCL2+, Cyclin D1−	BCL2 trans+ve and *BCL6* unbalanced trans+ve by FISH	

C‐bx, needle core biopsy; E‐bx, excision biopsy; n/a, not available; trans+ve, translocation positive; trans−ve, translocation negative.

Case 2: A 57‐year‐old female presented with acute small intestinal obstruction, and resection of the involved small intestine showed EMZL (Table 1). The patient was then treated with four cycles of rituximab to complete metabolic remission. Forty‐one months later, the patient had peritoneal deposits, and a biopsy of peritoneal LN displayed FL (grade 1–2). Interphase fluorescence *in situ* hybridisation (FISH) analyses demonstrated *BCL2* translocation in the peritoneal LN. BIOMED clonality analyses showed an identically sized clonal product with *IGK*‐B between the two lymphomas. The patient was treated with obinutuzimab‐bendamustine but unfortunately died of COVID after two cycles of the treatment.

Case 3: A 58‐year‐old male complaining of dysphagia and gastric biopsy showed EMZL (Table 1). The patient was treated with *Helicobacter pylori* eradication, then local radiotherapy (a total of 24 grey in 12 fractions), and achieved complete remission. Twenty‐six months later, the patient developed enlarged neck LN and tonsils. A LN biopsy showed FL (grade 3A). Interphase FISH showed no evidence of *BCL2* and *BCL6* translocation. He was treated with six cycles of rituximab‐bendamustine, achieved complete remission, and then underwent 12 cycles of rituximab maintenance.

Case 4: A 55‐year‐old male originally had bilateral FL of the conjunctivae and was treated with local radiation therapy to the left eye (right eye not treated as asymptomatic; Table 1). An endoscopy of the gastrointestinal tract was performed to investigate his anaemia, and histological examination of the biopsies showed gastric EMZL. Due to progressive disease with splenomegaly and lymphadenopathy above and below the diaphragm, further biopsies were performed. A biopsy of the left enlarged axillary LN revealed ISFN, and a further biopsy of a right buttock soft tissue mass showed FL. Retrospective analyses of these biopsies by interphase FISH showed *BCL2* translocation. There were no treatments for this patient between the different biopsies.

Case 5: An 88‐year‐old male had a right thigh subcutaneous mass, and an excision biopsy showed EMZL (Table 1). Forty‐eight months later, the patient developed enlarged right inguinal LN, and a biopsy showed FL grade 3A with both *BCL2* and *BCL6* (unbalanced) translocations by interphase FISH.

### Genetic investigations

Please refer to Supplementary materials and methods for details.

Additional interphase FISH for *BCL2* translocation and clonality analysis of the rearranged *IG* genes were performed if not done in routine histological diagnosis.

Tumour cell‐rich areas (>30%) from formalin‐fixed paraffin‐embedded tissue sections were microdissected and subjected to DNA extraction and next‐generation sequencing (NGS) of 278 lymphoma genes [11, 12].

## Results and discussion

**Figure 2:**
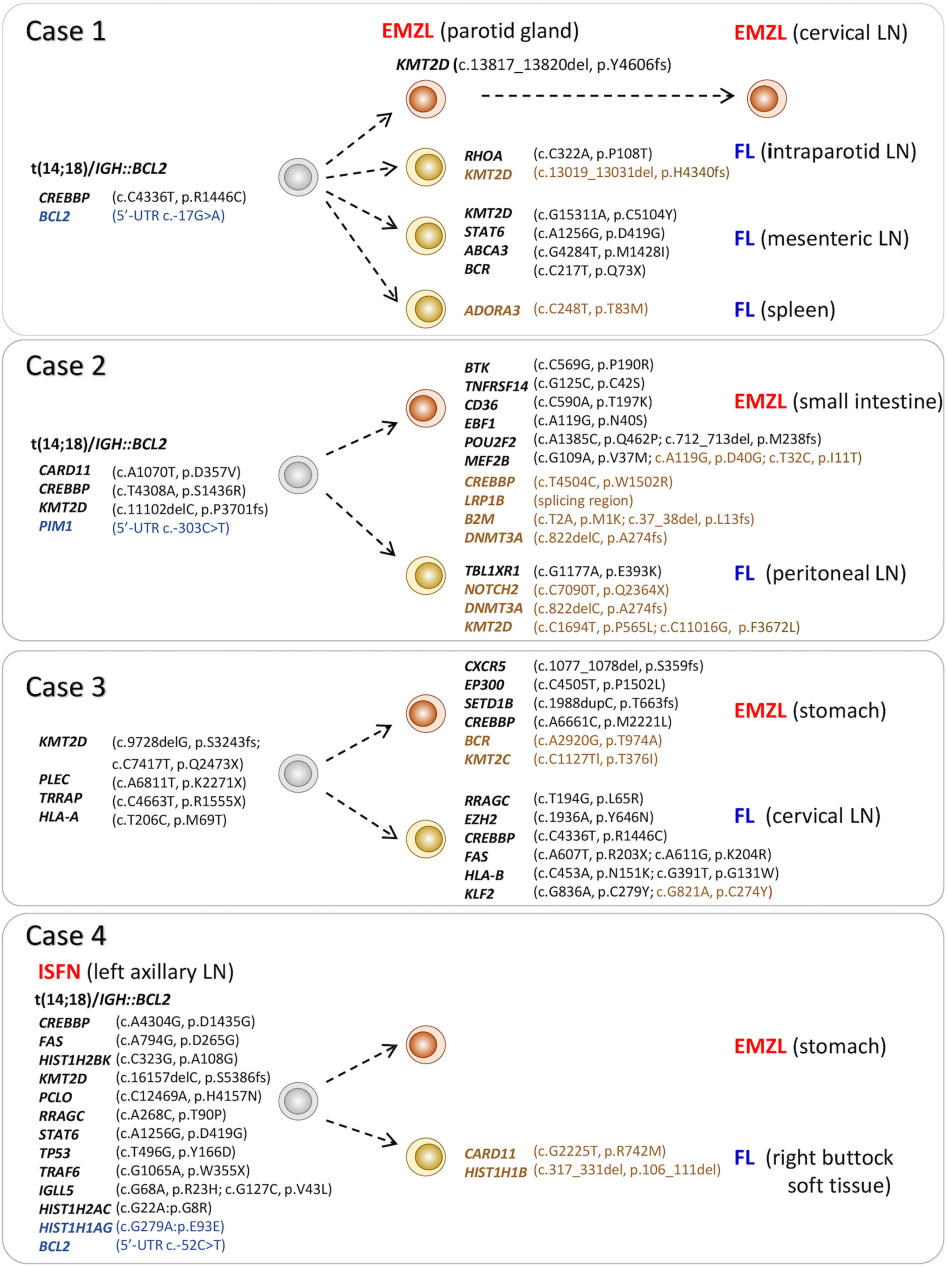
Divergent evolution of metachronous EMZL and FL. All somatic genetic changes, including variants in 5’‐untranslated region (UTR), are included in the phylogenetic illustration. Synonymous changes are shown in blue text, subclonal changes in brown. LN: lymph node; ISFN: *in situ* follicular B‐cell neoplasia.

The histopathology, immunophenotype, *BCL2* translocation, and *IG* gene rearrangement data are summarised in Table 1. Using mutational analysis of the paired EMZL and FL through NGS of 278 genes, it was possible to depict their evolutionary history in each case (Figure 2, Table S1, Figures S1 & S2).

In Case 1, all EMZLs of the parotid glands and cervical LN and FLs of the intraparotid LN in the second parotid biopsy, mesenteric LN, and spleen shared one common *CREBBP* mutation and a *BCL2* variant (Figure 2) but harboured variable numbers of distinct variants. All three EMZL biopsies harboured a unique *KMT2D* mutation but showed no mutation difference among themselves despite their marked intervals (12–18 months). In contrast, all three FLs showed a distinct mutation profile with two and four unique clonal mutations in the intraparotid LN of the second parotid biopsy and mesenteric LN respectively, but no further clonal mutation in FL of the spleen.

We reanalysed the rearranged *IGH* gene sequence from the previous study [10]. The CDR3 sequence (ARNGSHFDY) harboured an N‐glycosylation site, a common finding in FL, while the use of IGHV3‐7*01F and a short CDR3 length are features of rheumatoid factor [13]. It is highly likely that the BCR expressed by the lymphoma cells is autoreactive, and this may cause chronic BCR signalling, thereby contributing to lymphomagenesis.

In Case 2, the EMZL and FL shared four common clonal somatic variants and also harboured seven and one unique clonal mutations respectively (Figure 2). Similarly, in Case 3, the EMZL and FL displayed five common mutations, but four and eight unique clonal variants respectively (Figure 2). In Case 4, the EMZL and FL shared 14 somatic variants, which were also detected in the left axillary LN containing ISFN lesions (Figure 2). Interestingly, there was no major difference in the mutation profile between the EMZL and FL, with the exception of two subclonal changes (*CARD11* c.G2225T, p.R742M; *HIST1H1B* c.317_331del, p.106_111del) exclusively in the FL specimen.

In contrast, Case 5 lacked any shared mutations between the EMZL and FL (Supplementary material, Figure S1). This, together with the analyses of the rearranged *IG* genes, showed that the two lymphomas were clonally unrelated.

The common and unique mutation patterns between EMZL and FL in Cases 1–4 clearly indicate that these different lymphomas developed independently from a common premalignant B‐cell population, with evidence of *BCL2* translocation in three cases. Such a finding on divergent evolution cannot be extrapolated by routine analyses of *BCL2* translocation and *IG* gene rearrangements, although this provides information on a clonal relationship.

Cases 1–3 also showed subclonal mutations in both EMZL and FL. A proportion of these subclonal changes involved the genes, such as *KMT2D*, *CREBBP*, *MEF2B*, and *KLF2*, which are affected by the off‐target activities of somatic hypermutation machinery [14, 15]. It is possible that these subclonal changes were acquired during clonal expansion of FL lymphoma cells in the B‐cell follicle or follicular colonisation by EMZL cells.

Apart from the presence of *IGH::BCL2*, the mutations seen in FL among the cases investigated showed a classic mutation profile of FL, involving epigenetic regulators (*KMT2D*, *CREBBP*, *EZH2*) and mTORC1 (*RRAGC*) and JAK/STAT (*STAT6*) signalling. It is unclear what determines that a B‐cell carrying a *BCL2* translocation, the genetic hallmark of FL, will follow the trajectory of EMZL development. There were no further mutations in EZML within this study, which involved genes with well‐established roles in marginal zone B‐cell differentiation. Of note, there were no, or few additional, mutations detected in the EMZL of Cases 1 and 4, although it is not possible to exclude the possibility of other rare genetic changes beyond the 278 lymphoma genes investigated. Nonetheless, studies of ISFN show that *BCL2* translocation‐positive B‐cells can actively transit B‐cell follicles, migrating from one LN to another [2]. While transiting, the *BCL2* translocation‐positive B‐cells may encounter a niche that sustains their clonal expansion and transformation outside B‐cell follicles, together with genetic changes. Such niches may include critical microenvironments for relentless BCR stimulation and T‐cell help, which play a crucial role in EMZL development.

**Figure 3:**
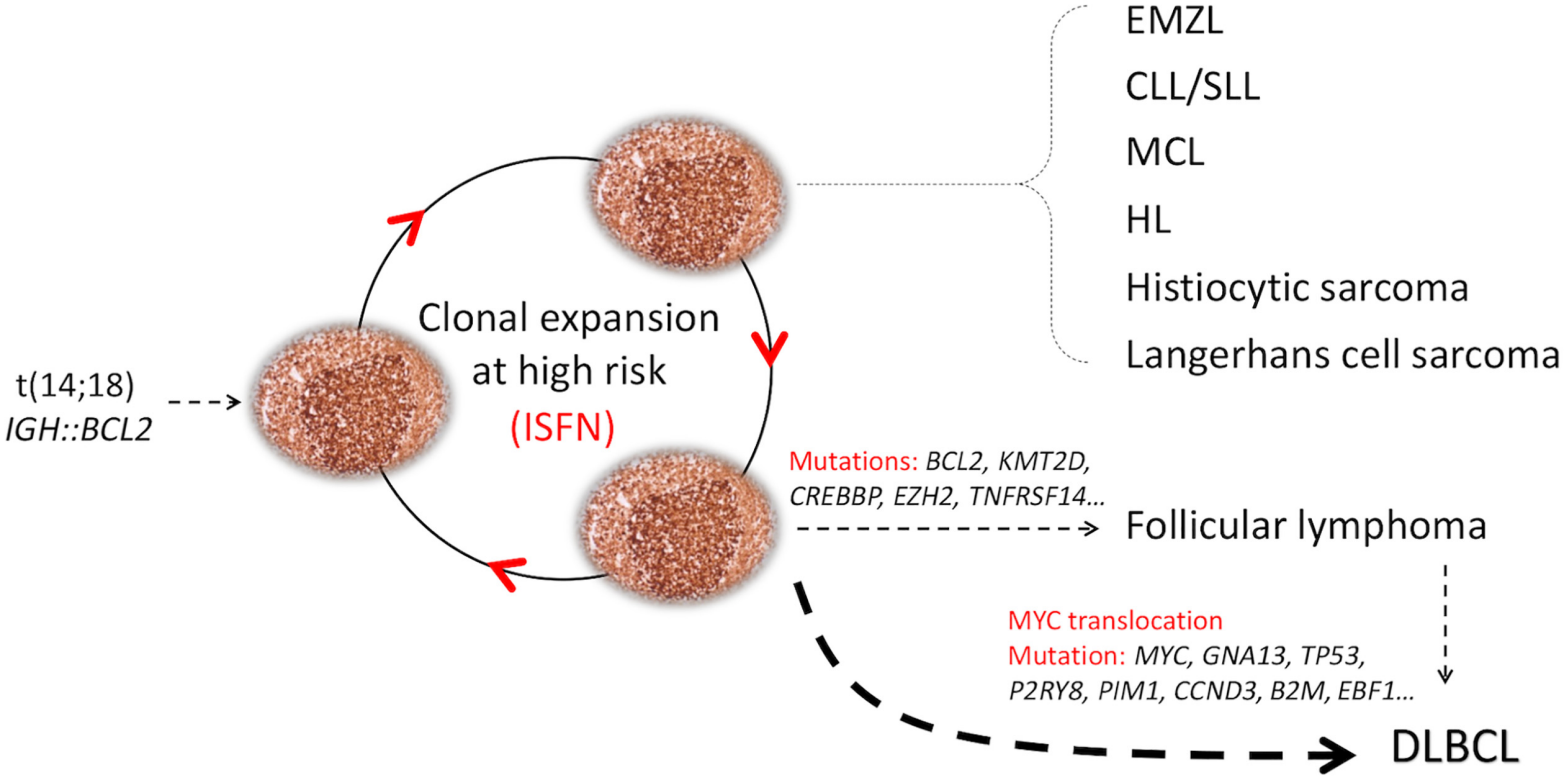
Multi‐malignant potential of *IGH:BCL2*‐positive cells. *IGH::BCL2*‐positive B‐cells, like reactive B‐cells, can readily undergo the GC reaction and transit from one B‐cell follicle to another, and thus are at a high risk of acquiring genetic changes, conferring multi‐malignant potential. CLL/SLL: chronic lymphocytic leukaemia/small lymphocytic lymphoma; MCL: mantle cell lymphoma; HL: Hodgkin's lymphoma.

A number of previous studies reported metachronous/synchronous FL and other lymphoid malignancies, including Hodgkin's lymphoma [16, 17, 18] (Figure 3). In each of these scenarios, the clonal link between the two different tumours was suggested by analyses of the *IGH::BCL2* fusion and/or rearranged *IG* genes. In one case of synchronous FL and histiocytic sarcoma, mutation analyses demonstrated that the histiocytic sarcoma originated from a common *IGH::BCL2*‐positive premalignant cell population [19]. Furthermore, *BCL2* translocation has also been seen in chronic lymphocytic leukaemia, albeit infrequently [20]. Finally, the majority of transformed FL, i.e. diffuse large B‐cell lymphoma, originated from their commonly related *IGH::BCL2* positive premalignant cell population rather than progressing directly from the preceding FL [6, 7, 8, 21]. These findings indicate the plasticity and diverse neoplastic potential of the *IGH::BCL2*‐positive premalignant cells.

*IGH::BCL2* causes constitutive overexpression of BCL2, an inhibitor of apoptosis, and this enables B‐cells to evade apoptosis while undergoing expansion during the GC reaction. *IGH::BCL2*‐positive B‐cells, like reactive B‐cells, readily undergo antigen affinity maturation and transit from one B‐cell follicle to another. FL may show marginal zone and plasmacytic differentiation and could manifest in the interfollicular region, even leading to a diffuse growth pattern. In this context, the findings of the multiple malignant potential of *IGH::BCL2* would be expected given that BCL2 functions as a universal apoptosis inhibitor regardless of cell lineage and differentiation stage and has no recognised role in blocking B‐cell differentiation.

## Author contributions statement

MMT, ZC, FC, JM and MQD designed the experiments and collected and analysed the data. AW contributed cases and pathological assessments. MQD, MMT and AW wrote and prepared the manuscript. MQD and AW designed and coordinated the study. All authors commented on the manuscript and approved its submission for publication.

## Supporting information


**Supplementary materials** **and methods**
**Figure S1.** Mutational profile of EMZL and FL in case 5
**Figure S2.** Average depth of reads of all cases analysed. Specimens with suboptimal DNA quantity and/or quality were investigated by targeted NGS in duplicates (referred to in Supplementary materials and methods).

**Table S1.** Variants detected by targeted next‐generation sequencing

## Data Availability

All experimental data related to this study are presented in the figures, tables, and supplementary figures and tables of the manuscript.
