# Supplementary material for: Divergent evolution of metachronous follicular lymphoma and extranodal marginal zone lymphoma of mucosa‐associated lymphoid tissue from a common precursor
Source: J Pathol. 2023 Jun 22;261(1):11–8. doi: 10.1002/path.6143 (PMC10952687; doi:10.1002/path.6143)
Supplement: Supplementary file 1 — Supplementary materials and methods Figure S1. Mutational profile of EMZL and FL in case 5 Figure S2. Average depth of reads of all cases analysed. Specimens with suboptimal DNA quantity and/or quality were investigated by targeted NGS in duplicates (referred to in Supplementary materials and methods). [file PATH-261-11-s002.docx]

**Divergent evolution of metachronous follicular lymphoma and extranodal marginal zone lymphoma of mucosa-associated lymphoid tissue from a common precursor**

M-M Tzioni *et al. J Pathol* <https://doi.org/10.1002/path.6143>

**Supplementary materials and methods**

**Supplementary Figure S1,S2**

**Supplementary Table S1 is provided as a separate Excel file.**

**Supplementary materials and methods**

Reference numbers refer to the main text list.

**Tissue materials and DNA extraction**

The use of archival tissues for research was approved by the ethics committees of the institutions involved. Formalin-fixed paraffin-embedded (FFPE) tissue biopsies were available in each case. Additional interphase florescence *in situ* hybridisation (FISH) was performed to detect *BCL2* translocation if not done in routine histological diagnosis using the Vysis LSI *BCL2* break-apart FISH probe (Abbott Molecular, Des Plaines, IL, USA) [22]. Tumour cell-rich areas (>30%) were microdissected from FFPE tissue sections and subjected to DNA extraction using the QIAamp DNA Micro Kit (QIAGEN, Hilden, Germany). The quality of DNA samples was assessed by PCR of variably sized genomic fragments [11].

**B-cell clonality analysis**

This was carried out by analyses of the *IG* gene rearrangements by adopting the BIOMED-2 PCR assays, followed by NGS using the Illumina MiSeq platform [2].

**Targeted next-generation sequencing**

The target panel includes a total of 278 genes and was designed for mutation profiling analysis of MZL, FL, and diffuse large B-cell lymphoma. A total of 200 or 20 ng of FFPE tissue DNA was fragmented using the Covaris E220 Focused Ultrasonicator (Covaris, Woburn, MA, USA) or restriction enzymes respectively. For each DNA sample, an indexed library was prepared with the xGen UDI-UMI indexes (IDT, Coralville, IA, USA) using the TWIST protocol and then pooled for target enrichment using TWIST probes (South San Francisco, CA, USA). The enriched DNA targets were amplified by PCR and pooled libraries were sequenced using the Illumina NextSeq 2000 platform (2×100 bp paired-end sequencing protocol) (San Diego, CA, USA). The sequence data analysis, variant calling, and filtering were performed as described in our previous studies [12,21]. The average depth of reads obtained is shown in supplementary material, Figure S2.

For DNA samples with suboptimal quality (PCR amplification of genomic fragment ≤200 bp) or inadequate quantity (using enzymatic fragment protocol), targeted sequencing was carried out in duplicate and only variants detected by both replicates were considered as a true change.

**Supplementary Figures S1,S2**


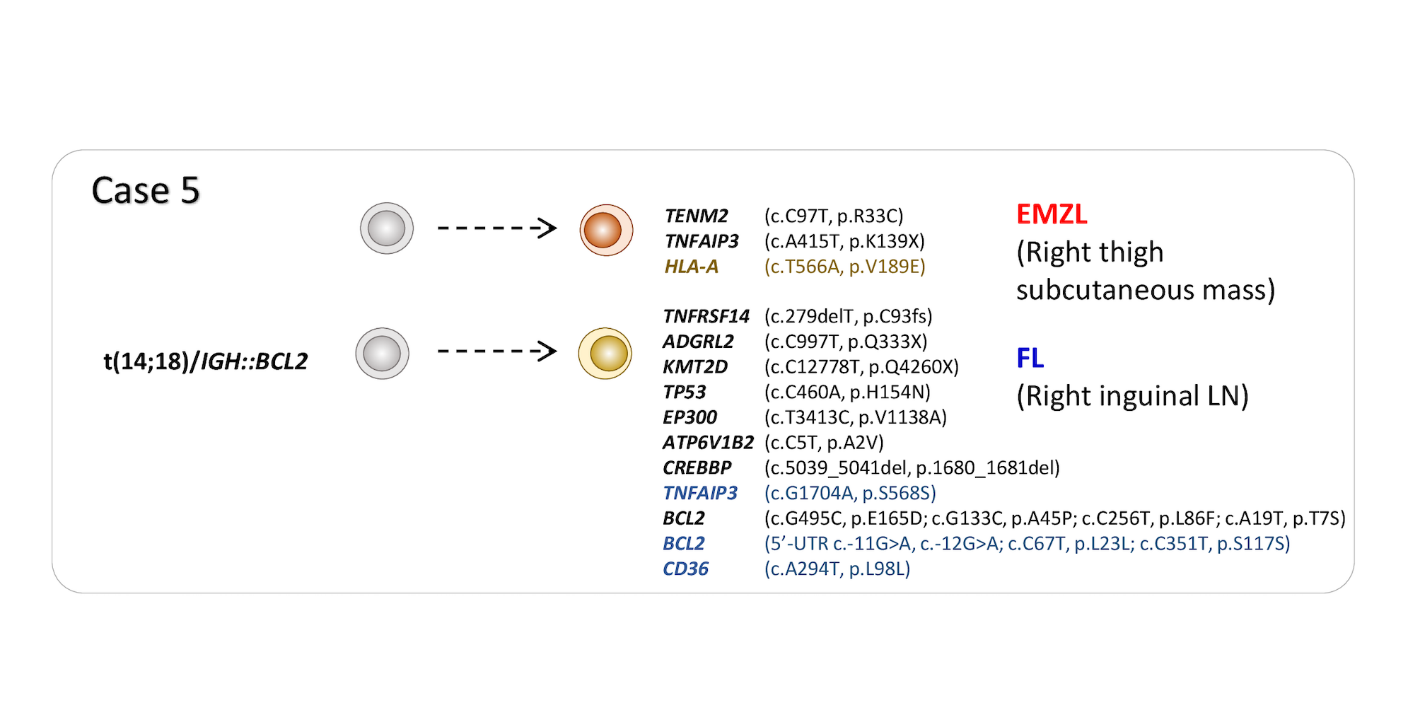


**Figure S1**. Mutational profile of EMZL and FL in case 5.


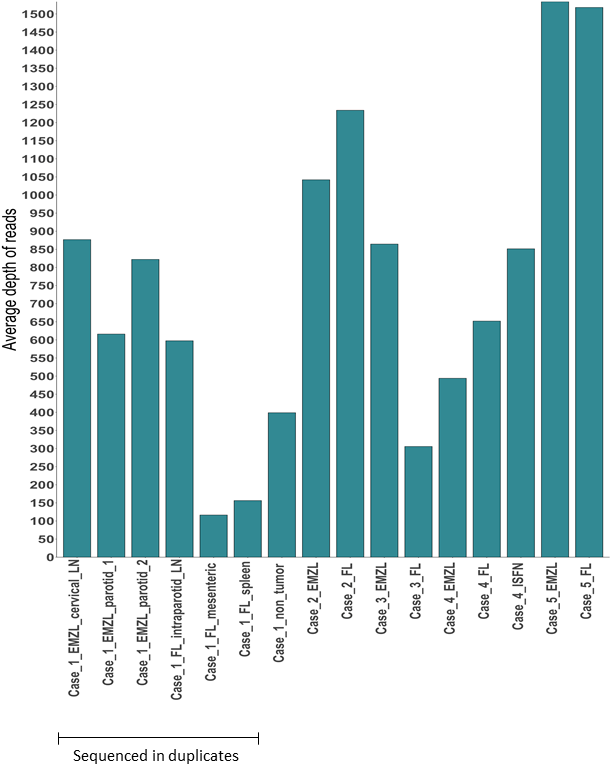


**Figure S2.** Average depth of reads of all cases analysed. Specimens with suboptimal DNA quantity and/or quality were investigated by targeted NGS in duplicates.
